# Evaluating the quality, reliability and readability of digital and artificial intelligence resources for adults with cancer who have significant caregiving responsibilities for children

**DOI:** 10.1371/journal.pdig.0001493

**Published:** 2026-07-09

**Authors:** Rohan Anand, Cherith J. Semple, Lisa Strutt, Sally Paul, Jeffrey R. Hanna

**Affiliations:** 1 Institute of Nursing and Health Research, Ulster University, Belfast, United Kingdom; 2 South Eastern Health and Social Care Trust, Ulster Hospital, Dundonald, United Kingdom; 3 Lisa Strutt Leadership and Coaching, Belfast, United Kingdom; 4 Department of Social Work and Social Policy, University of Strathclyde, Glasgow, United Kingdom; The Chinese University of Hong Kong, HONG KONG

## Abstract

Families often report searching the internet for guidance on how best to support children when a significant adult has cancer. This study aimed to identify and evaluate the quality, reliability, readability and content of websites, videos, and artificial intelligence (AI) resources available to adults with cancer who have caregiving responsibilities for children. Online platforms were searched using 10 phrases across Google web, YouTube, TikTok and four AI platforms. The mDISCERN instrument assessed reliability and quality, GQS assessed overall quality, and the NHS Medical Document Readability Tool assessed readability. Quantitative differences between sources were determined using pairwise analysis. Google web had significantly higher quality and reliability compared with AI and TikTok sources, with mean mDISCERN and GQS scores of 3.74 and 3.72, respectively. AI-generated resources showed lower mean mDISCERN and GQS scores of 2.77 (*P* < .05) and 2.32 (*P* < .05), respectively. TikTok videos had lower scores of 2.73 (*P* < .05) for mDISCERN and 2.49 (*P* < .05) for GQS. Estimated reading time was significantly longer (*P* < .05) for Google web (11:45mins) compared to AI (02:09mins). However, reading age did not differ (*P* = .31) at 15.09 years and 15.17 years respectively. There was a lack of accessible and inclusive resources for non-nuclear families, adults with neurodivergent children, culturally and ethnically diverse populations and families at end of life. Although Google web resources demonstrated higher overall quality and reliability, written resources across platforms often exceeded recommended reading levels, which may represent a significant health equity concern for individuals with lower health literacy and families experiencing deprivation. AI presents an opportunity whereby a single high-quality and evidence-based resource can be rapidly adapted into multiple formats, reading levels, languages and be culturally relevant. Future resources may benefit from co-production using a trusted, regulated, and centralised information hub, with supportive collaboration between health and social care professionals and technology providers.

## Author summary

This study looked at online platforms to help adults with cancer who have children (<18). While family-centred terminology is used conceptually throughout, the focus of this study specifically relates to adults with cancer who have caregiving responsibilities for dependent children. We searched online platforms to see how useful they were to help families as they support children during the cancer experience. The platforms included Google websites, artificial intelligence (AI), YouTube and TikTok. Resources were reviewed for the quality of advice they provided. Resources included websites, videos and AI responses. We found that websites found from Google had the best quality advice for adults. This was also mostly from trusted sources. AI and TikTok had poorer quality guidance to help adults with cancer. YouTube had some quality guidance. We found that written resources were likely difficult to read for most adults. We also found little information for diverse families. We suggest future online resources should be created together with health professionals, technology companies and patients. This will provide high quality and trusted guidance. This will better help all adults with cancer who have children.

## Introduction

Across the cancer trajectory, from diagnosis through to end of life, many families are often unsure how best to support dependent children (<18) when an adult with significant caregiving responsibilities has cancer. [1,2] Globally, 20% of adults of a parenting age are living with a cancer diagnosis [3] with an estimated one-in-20 children experiencing the death of a caregiving adult before adulthood. [4,5] Despite children’s desire to be involved in the cancer experience, they are often the ‘forgotten voice’ and excluded from key conversations and decisions within the family. [1,6,7] When information surrounding a significant adult’s cancer diagnosis or prognosis is withheld from children, they are predisposed to a greater risk of adverse mental health outcomes such as increased anxiety or depression. [8–10] Furthermore, children unprepared for the expectant death of a significant caregiver with incurable cancer are at increased risk of criminality and substance use, a decline in education, and greater involvement with psychiatry in later life. [11,12] Consistent with Family Resilience Theory, [13] clear and honest communication with children when a significant caregiver has cancer helps with maintaining and sustaining important relationships and mediating the risks of adversity.

There is limited information available for adult caregivers for children often desire instrumental support and guidance from health and social care professionals (HSCPs) on how best to prepare children across the cancer experience. [14] Despite being well positioned, many HSCPs feel ill-equipped to provide this important aspect of family-centred cancer care. [14–16] When support from HSCPs does not holistically address the needs of families, adult caregivers often report self-searching the internet for guidance on how best to support the children. [17,18] These include Google web searches, which account for over 90% of all internet searches, [19] with over 80% of people with cancer searching the internet for advice and guidance. [20,21] The reasons for people with cancer searching the internet for health-related information can include curiosity, a lack of adequate information from their healthcare team, a lack of trust of the information given by HSCPs or dissatisfaction with the care provision from HSCPs. [22,23] Other factors can include seeking reassurance and gaining empowerment [21].

Internet searching by patients seeking health-related guidance and support has been well established but lacks evaluation for reliability and quality. [24] Generative artificial intelligence (AI) is increasingly being accessed by people with cancer for health-related information. [25,26] It is estimated that there is around 300 million weekly active users on ChatGPT. [27] In addition, video resources are increasingly being used to share health-related content by healthcare providers, as well as patients sharing their lived-experience through social media platforms. [28,29] Given increases in searching for health-related information from a range of digital resources, including AI, it is important to assess the quality and reliability of these resources. [20,30] This is especially imperative for current social media platforms such as TikTok, as well as chat-bot style AI platforms; which have been readily accessible for the public in 2018 and 2023, respectively.

Despite the rapid expansion of digital resources and AI within healthcare contexts, the readability of the written content can contain complex terminology and sentence structure. [31] This drives health inequalities and does not serve the majority of the population. [32] These platforms require formal assessment to understand the applicability of information retrieved, especially for families in deprivation. Furthermore, evidence suggests that levels of literacy are lower amongst people living in deprivation [33] and are even lower for people living in low-and-middle income countries. [34] Overall, pre-existing barriers related to low literacy and deprivation contribute to poorer standards of cancer care among individuals living with cancer in hardship. [35] Alongside this, cancer diagnoses, and cancer-related death rates are significantly higher for families living in deprivation. [36] Deprivation is arguably the most influential factor forging difference in both cancer outcomes and experience of care. [32,37] There is a need to ensure that digital health-related information and guidance provided is understandable, accessible and of high quality for everyone, regardless of their background.

Therefore, this research aims to determine the suitability, quality, readability and content of digital resources when an adult with significant caregiving responsibilities for children (<18) has cancer.

### Objectives

Within our population of interest, namely, adults with cancer who are supporting children (<18), the objectives of this study were to:

1. identify and assess the quality and reliability of websites, videos and artificial intelligence (AI) resources.
2. assess the readability of websites and AI text resources in relation to estimated reading age and reading time.
3. explore the content of websites, videos, and AI resources for use across the cancer continuum.
4. explore the accessibility of the content of website, video, and AI resources for use across diverse populations.

## Methods

This cross-sectional study used a systematic scoping search and a comparative assessment of the suitability of available digital information for adults with cancer who have significant caregiving responsibilities for children. The search strategy, platform selection and research plan was developed by the research team and refined through patient and public involvement engagement (PPIE) input. This included adults with lived experience of cancer who have significant caregiving responsibilities for children (n = 3), a bereaved parent (n = 1) and a cancer nurse specialist (n = 1). This study including all searches, extractions and quality assessments took place between July 2025 and October 2025.

### Sources

Three sources were used to search for digital resources, to include Google web searches, YouTube and TikTok. Four generative AI platforms were used to produce AI responses, including Google AI (Gemini; version undisclosed by company), ChatGPT (Version GPT-4o), DeepSeek (V3.1) and WhatsApp (Meta AI; Llama 4). These resources were selected as it is estimated that over 80% of people with cancer use internet sources to search for cancer-related information. [20,21] Alongside this, these sources have not been compared within a single study before. [38,39] and were advised as the most appropriate platforms by the study’s PPIE group.

### Inclusion and exclusion criteria

Study inclusion and exclusion criteria were developed to ensure only eligible resources were included for the study. This is presented in Table 1.

**Table 1:** Inclusion and exclusion criteria.

	Inclusion Criteria	Exclusion Criteria
**Participants**	- Adults (aged ≥18) who have cancer and have a significant caregiving responsibility* for dependent child (<18). *This includes parents, as well as other significant relationships with children such as grandparents, aunts, uncles or legal guardians.	- Adults with other life-threatening or life-limiting illnesses that are not cancer. - Individuals under the age of 18 with cancer. - Resources targeted for children to self-guide when their significant adult caregiver has cancer. - Resources targeted for HSCPs.
**Resource format**	- Text-based websites, videos or generative AI with no word limit and no time limit on YouTube. - Published in the English language.	- Blogs, chatrooms, forum discussion sites as well as booklets, leaflets, books, hyperlinks, or adverts included within web pages that are aimed for printing. - TikTok videos that were over 4 minutes in length. - Non-text resources identified through Google websites that included video and audio. - Non-English resources.
**Content**	- Content across the cancer trajectory (diagnosis, treatment, end of life, dying).	- Non-cancer related content

### Search strategy

A search strategy was developed combining a total of ten keyword phrases (Table 2). Keyword phrases were initially identified by the authors who are experienced oncology and palliative care professionals and researchers, as well as a bereaved adult who has significant caregiving responsibilities for children (LS). The ten keyword phrases were then verified by the PPIE group who considered the searches as the most relevant, sensitive and appropriate terms that may reflect internet searches entered by adults with cancer who have caregiving responsibilities for children. The ten keyword searches (Table 2) were completed on all platforms separately and not combined. The Boolean operator ‘OR’ was used to combine similar keyword phrases on Google Web searches. All searches were completed by one author (RA or LS). Each keyword phrase search was completed on a new incognito browser to reduce the impact of internet cookies and results based on prior search history. This was considered appropriate as this would not generate individualised results based on the study author’s profile. No follow up prompts were used for any platforms following searches.

**Table 2:** Keyword phrase searches used to identify resources.

Keyword phrase one: ‘How to tell children about cancer’
Keyword phrase two: ‘How to tell teenagers about cancer’
Keyword phrase three: ‘How to support children when I have cancer’
Keyword phrase four: ‘How to support teenager when I have cancer’
Keyword phrase five: ‘How to tell children about cancer treatment’
Keyword phrase six: ‘How to tell teenagers about cancer treatment’
Keyword phrase seven: ‘Talking to children about dying with cancer’
Keyword phrase eight: ‘Talking to teenagers about dying with cancer’
Keyword phrase nine: ‘How to support children when someone is dying with cancer’
Keyword phrase ten: ‘How to support teenagers when someone is dying with cancer’
*NB: For Google web searches, the Boolean operator ‘OR’ was used to combine phrase: one OR two, three OR four, five OR six, seven OR eight, nine OR ten.*

### Screening and selection

For the Google web searches, the first 50 results from each paired keyword search were screened against eligibility criteria (n = 250). For each AI source, the singular generated response for each individual keyword search was screened against eligibility criteria (n = 40). For YouTube and TikTok, the first 20 results from each individual keyword search were screened against eligibility criteria for each platform respectively (n = 200 for each). These numbers were deemed appropriate to find the most relevant resources as research highlights the general public usually view only the first section of results when internet searching. [40] Also, these parameters would identify a significant number of resources applicable to our population of interest and so provide adequate information saturation. Evidence highlights that shorter TikTok videos (<60seconds) are more likely to receive immediate attention and higher completion rates, [41] though videos between one and three minutes are more effective for advice giving. [42,43] To promote sensitivity for inclusion of TikTok resources, videos were included that were up to four minutes in length. With the exception of TikTok, no account creation was needed for completing searches.

Data extraction, content mapping, reliability, readability and quality assessments were primarily completed by one researcher (RA or LS). To enhance accuracy and consistency, 10% of extracted data, content mapping, mDISCERN scoring, GQS scoring, and readability assessments were independently checked by a second researcher (JRH), with greater than >90% agreement observed across reviewed components [44,45]. Disagreements were resolved with arbitration with a third researcher (CJS).

### Data extraction

Data were extracted using specifically designed data extraction tools for each source on Microsoft Excel. Data were extracted related to the following items: author accreditation, type of author, type of resource, resource length, applicability across cancer trajectory (diagnosis, during treatment, end of life, dying), country of origin, child developmental age relevancy of information (all ages, 0 – 5 years, 6 – 11 years, 12 – 18 years), resources analytics (date uploaded, number of views, likes, shares, comments, YouTube health accreditation), and inclusivity/accessibility (defined as translated to other languages, cultural/ethnic aspects, families living in deprivation, families from non-traditional/non-nuclear family units, children with additional needs such as neurodivergence).

The content within the resources were mapped to a matrix based on the key needs of families across the cancer trajectory (see S1 File). [1,2,6,18] This included the needs of families when a significant adult caregiver of children: (1) receives a cancer diagnosis, (2) is navigating treatment, (3) is managing the end of life trajectory (defined as an incurable cancer diagnosis and where death is expected within 12-months and (4) is navigating the final weeks and days of life (defined as the dying period). [46] The matrix was developed by JRH and CJS who are experienced clinical-academics and are subject experts in family-centred cancer care.

### Reliability and quality assessments

The quality and reliability of the resources were assessed using a modified DISCERN (mDISCERN) which comprises of five questions: [47] 1. Is the response/video clear, concise, and understandable, 2. Are valid sources cited, 3. Is the response provided balanced and unbiased, 4. Are additional sources of information listed for patient reference, 5. Does the response address areas of controversy/uncertainty. A ‘yes’ response denoted a value of 1. Total score for each resource was scored out of a maximum of 5.

The Global Quality Score (GQS) was used to assess for overall quality of the whole resource. [47,48] GQS is a 5-point scale ranging from 1-5, with 1 indicating poor quality and 5 excellent quality. One statement of the following statements is applied to the resource; 1. Poor quality, poor flow, most information missing, not helpful for patients. 2. Generally poor, some information given but of limited use to patients. 3. Moderate quality, some important information is adequately discussed. 4. Good quality good flow, most relevant information is covered, useful for patients. 5. Excellent quality and excellent flow, very useful for patients. The higher the score the higher the quality of the resource. Both the mDISCERN and GQS scales were worded appropriately within the extraction sheets for the source type: website, AI response or video.

### Readability assessment

Google web Searches and AI were assessed for readability using the online NHS Medical Document Readability Tool, [49] that estimates reading age, reading time and calculates the number of words. It is primarily based on the Flesch-Kincaid system of measurement [50].

Data extraction, content mapping, reliability, readability and quality assessments were completed by one researcher (RA or LS). To enhance accuracy and consistency, 10% of extracted data, content mapping, mDISCERN scoring, GQS scoring, and readability assessments were independently checked by a second researcher (JRH), with greater than >90% agreement observed across reviewed components. Disagreements were resolved with arbitration with a third researcher (CJS).

### Data analysis

Key characteristics of the included resources were analysed according to country of source and the type of publisher/uploader. The four AI platforms were grouped together as they are all types of AI primarily based on large language models (LLMs). These are generative assistants that have technical similarities and large overlapping capabilities for tasks. [51] Quantitative differences in mDISCERN, GQS and NHS readability scores were determined using a pairwise analysis following histogram assessments for data normality. A Kruskal-Wallis test was first performed for any four-group analysis, followed by pairwise Mann-Whitney U tests with Bonferroni correction. Analysis was presented as mean, median and range to provide granularity and allow comparison. Inferential conclusions were based on rank-based analyses Significance level was *p* < .05. Quantitative analyses were performed in R Studio (2025.09.2; Build 418) with graphs produced in Microsoft Excel. Content relating to the cancer trajectory and content matrix within the included resources were explored and analysed as a designation manifest content analysis, which obtains the frequencies of similar words, groups or concepts and presented as percentages [52].

### Patient and public involvement and engagement (PPIE)

A synthesis of the findings was initially presented to a PPIE group (representatives identified earlier) and the project steering group. This study’s project steering group consisted of two people with lived experience, a palliative care consultant, a cancer family support and art therapy coordinator, a cancer social support specialist, an oncology nurse and a palliative care psychotherapist. Their input was sought to refine and verify the clinical recommendations and relevance for this population.

A synthesis of the findings was also presented to four technology industry experts in data engineering and AI. This was to refine and verify the technological recommendations and directions for future research and resources in this research and topic.

## Results

### Searches, screening and selection

**Fig 1:**
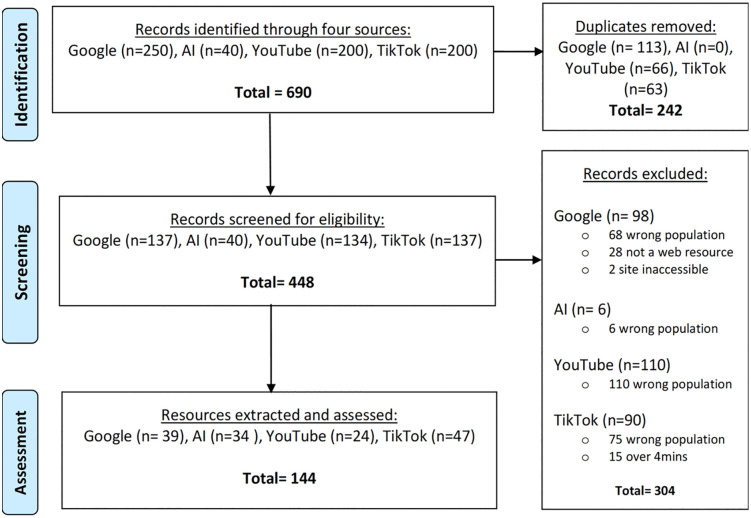
Flowchart diagram outlining the identification, screening, selection and assessment of identified records. AI: Artificial Intelligence.

Searches included a combined total of 690 records for screening. After the removal of duplicates and screening against inclusion criteria, a total of 144 resources met eligibility criteria and were included for analysis. This included 39 resources from Google web, 34 from AI responses, 24 from YouTube and 47 from TikTok. The full details relating to the identification, screening, duplicate removal and reasons for exclusion of records is contained within Fig 1.

### Overview of included resources

A total of 144 resources were included from the four sources: Google Web Searches (n = 39), AI (n = 34), YouTube (n = 24) and TikTok (n = 47). Resources were predominately from the United States of America (44%) and the United Kingdom (29%). Fifteen percent of resources were from Australia, Canada, China, Ireland, New Zealand, and South Africa. The origin of 13% of resources were unreported or unclear. Resources from the Google Web searches were predominately developed by charities (67%), with YouTube content predominately provided by a combination of charities (21%), industry (29%), academia (13%) or independent channels (17%). TikTok content was mostly provided by patients and families with lived experience (75%) with some content also provided by HSCPs (15%) and charities (6%). In relation to the publication date and the age of resources on the platforms, those on YouTube had a mean age of 6.21 years old. Google websites had a mean publication date of 3.17 years old and TikTok was the newest at 1.32 years old. In relation to video metrics on YouTube and TikTok, the median views, likes and comments were all statistically significantly higher on TikTok resources (*P* < .05). A total of 33% of YouTube videos were YouTube Health Accredited. Overall, 13% of the videos on YouTube were animated with none on TikTok. Full characteristics and viewing metrics of the included resources across Google Web Searches, AI, TikTok and YouTube are shown in Table 3.

**Table 3:** Resource characteristics according to source publisher, country of origin and video metrics.

*Characteristic*	Google (N = 39)	AI (N = 34)	YouTube (N = 24)	TikTok (N = 47)	*P-value* P_Y-T_
*Source Type % (n)*					
AI derived	0	100% (34)	0	0	–
Cancer patient/family	0	0	8.3% (2)	74.5% (35)	–
Charity	66.7% (26)	0	20.8% (5)	6.4% (3)	–
Digital media publisher	5.1% (2)	0	0	0	–
HSCPs	0	0	8.3% (2)	14.9% (7)	–
Independent YouTube channel not associated with any organisation	0	0	16.7% (4)	0	–
Medical benefit fund	2.6% (1)	0	0	0	–
Private Company	0	0	29.2% (7)	0	–
Private Healthcare Provider	7.7% (3)	0	0	0	–
Public/Academic Healthcare Provider	18% (7)	0	12.5% (3)	0	–
Talkshow	0	0	4.2% (1)	4.3% (2)	–
*Country of Source % (n)*					
Australia	7.7% (3)	0	4.2% (1)	0	–
Canada	2.6% (1)	0	4.2% (1)	0	–
China	0	29.4% (10)	0	0	–
Republic of Ireland	2.6% (1)	0	0	4.3% (2)	–
New Zealand	2.6% (1)	0	0	0	–
South Africa	2.6% (1)	0	0	0	–
United States of America	46.2% (18)	70.1% (24)	16.7% (4)	36.2% (17)	–
United Kingdom	35.9% (14)	0	74.9% (18)	19.1% (9)	–
Unknown	0	0	0	40.4% (19)	–
*Video metrics*					
Views (M, Med, R)	–	–	3980 (960; 1-60636)	36579 (2538; 457-809800)**	<0.05
Likes (M, Med, R)	–	–	104 (9; 0-1900)*	25181 (100;10-110000)	<0.05
Comments (M, Med, R)	–	–	8 (0; 0-134)*	1146 (7; 0-43700)	<0.05
Video duration mins (M, Med, R)	–	–	05:36 (03:45; 01:00-34:33)	01:44 (01:31; 00:12-03:49)	-***
YouTube Health Accredited % (n)	–	–	33.3% (8)	–	–
Animations % (n)	–	–	12.5% (3)	0	–

- = Comparison/data not applicable. AI = Artificial Intelligence, n = number, % = percentage, M = Mean Med = Median, R = Range, min = minutes, sec = seconds, P = P Value, Y = YouTube. T = TikTok. *YouTube likes (N = 23), comments (N = 22) due to hidden/restricted metrics on the platform. **TikTok views (N = 28), due to hidden metrics on the platform. *** Comparison not completed as TikTok data was restricted to only videos less than 4 minutes.

### Quality and reliability

Google Web resources demonstrated significantly higher quality and reliability compared with AI and TikTok sources, with mean mDISCERN and GQS scores of 3.74 and 3.72, respectively. AI-generated resources showed lower mean mDISCERN and GQS scores of 2.77 (*P* < .05) and 2.32 (*P* < .05), respectively, while TikTok videos had similarly lower scores of 2.73 (*P* < .05) for mDISCERN and 2.49 (*P* < .05) for GQS. These findings indicate that Google Web resources provide better overall quality and reliability relative to AI-generated content and TikTok video sources. YouTube videos had mDISCERN and GQS scores of 3.00 and 2.93, respectively which did not significantly differ from AI or TikTok and showed no clear difference from Google (*P* = .05). Across platforms, between-group differences were large for both outcomes, with ordinal eta-squared effect sizes of 0.178 for mDISCERN and 0.214 for GQS. Details of the mDISCERN and GQS scores are in Table 4.

**Table 4:** mDISCERN and GQS reliability and quality, and NHS readability assessment.

	Google (N = 39)	AI (N = 34)	YouTube (N = 24)	TikTok (N = 47)	*P*-value*					
***Assessment Criteria***					P_G-AI_	P_Y-T_	P_G-Y_	P_G-T_	P_AI-Y_	P_AI-T_
mDISCERN score (M, Med)	3.74 (4.00)	2.77 (3.00)	3.00 (3.00)	2.73 (3.00)	<0.05	1.00	0.11	<0.05	1.00	1.00
GQS score (M, Med)	3.72 (4.00)	2.32 (3.00)	2.93 (3.00)	2.49 (2.00)	<0.05	0.66	0.05^	<0.05	0.26	1.00
NHS readability score, estimated age years (M, Med, R)	15.09 (15.00; 13.4-17.8)	15.17 (14.35; 12.7-20)	–	–	0.31	**–**	**–**	**–**	**–**	**–**
NHS readability score, estimated reading time (mins:secs) (M, Med, R)	11:45 (06:08; 01:32-112:00	02:09 (01:32; 00:06- 05:02	–	–	<0.05	**–**	**–**	**–**	**–**	**–**
Number of words (M, Med, R)	3025 (1554; 475-28060)	586 (471; 41-1261)	–	–	<0.05	**–**	**–**	**–**	**–**	**–**

- = Comparison/data not applicable. M = Mean, Med = Median, R = Range, mins = minutes, secs = seconds, Y = YouTube. T = TikTok, AI = Artificial Intelligence, G = Google. * Kruskal–Wallis test first performed for 4 group analysis, followed by pairwise Mann–Whitney U test with Bonferroni correction. ^ result is at the significance threshold.

### Readability

The mean number of words contained in Google Web resources were statistically significantly higher at 3025 words compared to AI at 586 words (*P* < .05), with a large effect (r = 0.655). The estimated reading time was statistically significantly longer for Google Web resources with a NHS readability mean time of 11:45 mins compared to AI responses mean reading time of 02:09 mins (*P* < .05), with a large effect (r = 0.655). There was no significant difference (*P* = .31) in the mean NHS estimated reading age between Google Web (15.09 years) and AI resources (15.17 years), with a small effect size (r = 0.120). Details of the NHS readability assessment are reported in Table 4.

### Content across the cancer trajectory

**Fig 2:**
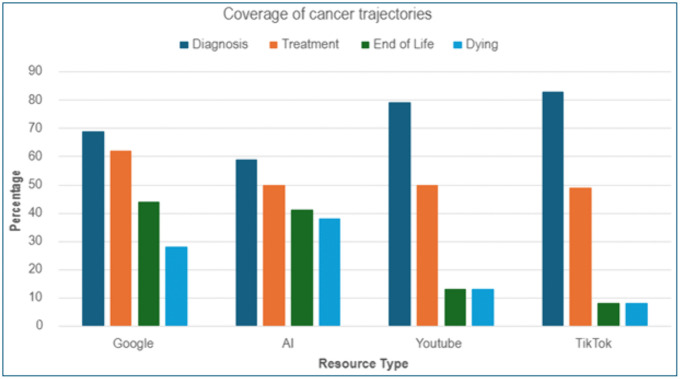
The percentage content coverage across the cancer trajectory (diagnosis, treatment, end of life, dying) together with accessible/inclusive information from the 144 resources. AI: Artificial Intelligence.

Across the cancer trajectory, it was identified that 72.9% of resources were relevant for the needs of families when an adult with significant caregiving responsibilities receives a cancer diagnosis, with 52.9% relevant when navigating treatment, 26.4% at end of life and 21.5% when the adult was dying. The full details across the sources are visualised as a clustered bar chart in Fig 2.

### Content in relation to equity, diversity and inclusion

Content related to aspects of inclusivity was 23.6% (previously defined as translated to another language, cultural/ethnic aspects, families living in deprivation, families from non-traditional/non-nuclear family units, children with additional needs such as neurodivergence). The full details of the content analysis showing specific items within each trajectory (diagnosis, treatment, end of life, dying) according to the matrix is contained within S1 File.

## Discussion

### Summary of findings

Traditional text-based website resources identified through Google web searches for adults with cancer with significant caregiving responsibilities for children had higher quality and reliability compared to TikTok’s short form video and AI outputs. YouTube videos did not statistically differ in quality and reliability when compared to Google, TikTok or AI, however, this was at the threshold of significance when compared to Google. This suggests that adults with cancer accessing websites via Google web searches are overall more likely to encounter materials of better quality and reliability. The reading age was similar between Google web and AI resources. However, the estimated reading time for Google web resources was 5–6 times longer compared to AI responses thus requiring longer attention from users. Overall, there was a lack of inclusive resources representative of family diversity, to include same-sex and/or single parent families, families living in deprivation, neurodivergent children, or aspects capturing cultural and ethnic diversity. Furthermore, when content of the resources was considered, there was a lack of available resources focusing on the end of life/dying period compared to diagnosis and treatment.

### Discussion of findings

Compared to AI text responses, resources identified through Google web searches were of a better quality and from more authoritative sources such as from HSCPs. This finding aligns with previous studies, in that the quality and reliability of traditional health websites developed by HSCPs often outperform AI. [53–55] For example, the usability and reliability of AI answers to clinical questions are often inferior to expert authored resources from credible sources. [56] Despite this, the estimated reading age for both Google web and AI searches was estimated at 15-years old. Although quality of health-related information is important, it is vital that the content can be understood by the population who it should benefit. [57] Whilst global literacy rates are on the rise, [58] levels of literacy are lowest amongst people living in deprivation. [33] Furthermore, the average reading age in English speaking developed nations is frequently lower than 15-years old, with recent estimates suggesting a mean of 9–11 years old. [34,59,60] Other studies have identified similar findings with readability of high-quality resources being greater than recommended reading levels. [61–63] This demonstrates that the higher quality information available through Google web may not be usable for many, and even more so for families who are living in deprivation or have low health literacy. This is in addition to other barriers to digital access such as limited digital connectivity due to cost and poor information retrieval skills. [64] This can lead to a risk of widening inequalities in cancer care.

Similar to other studies, YouTube resources were identified to be of better quality when compared with TikTok. [28,65–68] However, in this study this difference approached statistical significance and was accompanied by substantial variability. There was significantly more user engagement with TikTok resources. This may be due to the content on TikTok being largely created by patients with lived experience, as often people impacted by cancer have a desire to hear similar stories and can help individuals feel less isolated, normalise their situation, find hope and glean practical advice from others who understand their experience of navigating the cancer trajectory with their children. [18] It is possible that powerful algorithms optimise the attention of the person with cancer through highly emotive content. [39] Further explanations for higher engagement with TikTok as opposed to other sources, such as YouTube, may be attributed to the increase in the speed of dissemination across the platform, as well as regular viewing of short form video content. [69]Also, older platforms such as YouTube, which have been shown to contain credible videos on the topic of family-centred cancer care from leading healthcare experts, [70] are becoming increasingly less accessed by younger adults. [29] While TikTok may be considered as the ‘social media platform of the moment’, presenting another opportunity to engage with people who are impacted by cancer, this will likely change over time as newer social media platforms evolve. [71] Therefore, it is important that HSCPs understand how to enhance engagement with high-quality, evidence-based content not only on authoritative sources, but on platforms with which the public frequently engage with to provide health-related information and guidance, such as TikTok. Alongside this, there is a need to better understand how adults with cancer use non-authoritative content as sources of guidance such as TikTok and generative AI.

### Implications of findings

There is a need for the co-development of inclusive and accessible cancer resources across the cancer trajectory that recognise the diversity of family life, including those experiencing social and economic deprivation, which are also reflective of cultural and ethnic diversity. This would allow for greater health equity in family centred cancer care. [72] With ongoing advancements and investments to digital resources within cancer care, [73,74] especially AI, it is important that any new resources are validated to provide reliable, accurate and accessible information. Although AI has the potential to support families within cancer care, it is not a one-size-fits-all approach. [75,76] To produce tailored guidance for families, evidence suggests that it would be preferable for the system to ask specific prompts of the user, to ensure the health information provided is applicable, reliable, evidence-based and of high quality. [77] This may include prompting for the age of the children, any neurodivergence or special educational needs or perhaps specific details relevant to the socio-cultural context of the family. While many people with cancer may not be accustomed to searching AI with such high detail and specifics, AI platforms currently lack consistent clinical validation based on user prompts. [78] As a result, any question or phrase can be inputted by the user, regardless of applicability, and an output will be generated by the system that can sound convincing. [79] Consequently, people with cancer are obtaining information from digital and AI sources where powerful algorithms are shaping what content is provided, regardless of what the person actually needs [39].

Although traditionally the responsibility has been with HSCPs to provide reliable healthcare information to patients, over 80% of patients are accessing the internet for information relating to their cancer experience [20,21]. Hence, technology companies have a disproportionate control in the digital world as to what is accessible to large populations. [80] Technology companies have a responsibility to provide societal stewardship and promote digital health resources that are credible. [81] As AI-powered searches and LLMs become a more common first point of enquiry, future access to health-related information is also likely to shift away from traditional web searches toward summarised, conversational answers. [82,83] In the short-to-medium term, this increases the risk that people are provided information that sounds authoritative but is not clinically validated or accessible to their needs. [77,84] Such AI-generated health information can seriously affect users’ perceptions of their health and lead to decision-making with irreversible consequences. [85] Despite these potential drawbacks, AI presents a significant opportunity whereby a single high-quality and evidence-based resource can be rapidly adapted into multiple formats, reading levels, language and be made culturally relevant. [86,87] However, without appropriate review and quality checking, AI-adapted content may risk introducing inaccuracies, hallucinations, or culturally inappropriate information. To minimise these risks, future digital resources are co-created using a trusted, regulated, and centralised information hub or LLM agent, with supportive collaboration between HSCPs and technology companies, to ensure AI-driven digital ecosystems provided families with reliable information at the right time. [88] There has been recent development in this concept with the launch of ChatGPT Health. [89] However, such initiatives still require careful assessment of their quality, reliability and safety before they can be confidently recommended to families affected by cancer.

Approximately one third of YouTube videos in this study were YouTube Health Accredited; a process where uploaders are vetted through a third-party against reasonable eligibility criteria, [90] with the aim of directing people towards high-quality sources. If a ‘badging’ system were added to digital resources it could add credibility to resources deemed appropriate for patients, or in instances, those deemed inappropriate. This could ensure people with cancer are accessing high-quality information and guidance if more platforms implement a legitimate certification process for the information they provide.

### Strengths and limitations

This study compared a large number of resources from multiple sources, allowing for a comprehensive analysis which found a clear hierarchy of quality. The study used commonly accepted and validated tools to assess for reliability, quality and readability within video and text-based resources [47,48] and this supported comparisons of findings to previous studies in the discussion of findings. Some of the information and resources included from Google web searches and YouTube were published before generative AI was widely accessible, implying that AI generated content (text and video) is less likely to be used to develop content from most of these two sources. PPIE individuals added important insights and critique to the direction of research from study inception to preparation of this manuscript. This strengthened the quality of the search terms, type of sources searched, the context of results relating to the workings of AI and internet technology and also applicability of included resources for families.

Limitations are that searches were completed within one geographical location in a Western context, indicating a location and language bias towards resources from developed English-speaking countries with readability calculated using the NHS Readability Tool. Additionally, search terms may not account for the exhaustive list of words and phrases that an individual may use when searching the internet for information and guidance. [91] Similarly, the online platforms available to search is not exhaustive but represented what is likely to be used by patients as guided by the study PPIE group. Furthermore, patients may have accounts and substantial prior search history with platforms which may lead to highly individualised results including the use of AI prompts. Although quality assessments were completed by a single reviewer, the random validity checks found very high agreement. Whilst this study was able to assess user engagement across TikTok and YouTube, it was unable to assess the extent of and reason for engagement, and the extent to which engagement shaped understanding and behaviours across all platforms. More research is thus needed to explore the relationship between interaction and utility with online resources and engagement with children when a significant adult has cancer. Although this study showed distinctions between different sources, it is important to note that even resources from within a single source can vary in quality due to resource specific characteristics. [92] Additionally, some score differences may reflect the structural design of short-form video platforms, which may provide less opportunity to include detailed evidence or information sources. Whilst websites identified via Google web searches were of a higher quality to AI, not all search results were of the same quality from the four sources (TikTok, YouTube, Google web and AI). Additionally future research could assess trends over time. However, AI-generated responses may change over time due to ongoing model updates, refinement processes, and changes to platform safety filtering, meaning identical prompts may produce different outputs across time points.

## Conclusion

For adults with cancer who have caregiving responsibilities for children, Google searches demonstrated higher overall quality and reliability in content compared with AI-generated content and TikTok video sources, although substantial variability existed across platforms. While AI-generated responses were shorter and quicker to read, their quality scores were generally lower, highlighting the need for cautious use of AI-generated cancer information. Importantly, the high reading age required across written resources may represent a significant health equity concern, particularly for individuals with lower health literacy and families experiencing deprivation. While AI may offer opportunities to improve access to health information, AI-generated responses may lack the depth and contextualisation required to address the individual needs and circumstances of families affected by cancer. Additionally, there remains a lack of accessible and inclusive resources for non-nuclear families, neurodivergent children, culturally and ethnically diverse populations, and families at end of life. There is an urgent need for clinicians, technology companies, and policymakers to support the co-production of inclusive and accessible cancer resources using trusted, evidence-based and regulated approaches. These processes will promote availability of content that is accessible, culturally relevant, and responsive to the needs of families across the cancer continuum. Ultimately, this could help ensure children are supported across the cancer trajectory, promoting psychological growth and resilience both now and in the future.

## Supporting information

S1 File Extraction matrix for content analysis. Content Analysis graphs from the extraction matrix. References.
(DOCX)
