# Supplementary material for: Evaluating the quality, reliability and readability of digital and artificial intelligence resources for adults with cancer who have significant caregiving responsibilities for children
Source: PLOS Digit Health. 2026 Jul 9;5(7):e0001493. doi: 10.1371/journal.pdig.0001493 (PMC13347835; doi:10.1371/journal.pdig.0001493)
Supplement: S1 File — Content Analysis graphs from the extraction matrix. References. (DOCX) [file pdig.0001493.s001.docx]

**Supplementary Materials**

Contents

[Extraction Matrix for content analysis Based on contents within the Cancer trajectory at diagnosis, treatment, EoL and dying 2](#_Toc228453000)

[Content Analysis graphs from the extraction matrix 3](#_Toc228453001)

[References: 4](#_Toc228453002)

## Extraction Matrix for content analysis Based on contents within the Cancer trajectory at diagnosis, treatment, EoL and dying

| **Needs of parents at diagnosis** ^1-3^  - Advice and guidance on how to tell the children about the cancer diagnosis.  -Maintaining routines as best as possible.  - Advice on keeping key networks (e.g., schools) updated.  - Information on the reactions of children to a cancer diagnosis.  -   Information within the resource provides developmentally appropriate information. | **Needs of parents during treatment** ^1-3,4^  -   Advice and guidance on how to tell the children about cancer treatments and its effects.  - Guidance on how to involve the children throughout the cancer experience.  -Information within the resource provides developmentally appropriate information | **Needs of parents at end of life**^5-10^  - Advice and guidance on how to tell the children about the poor cancer prognosis.  - Guidance on how to involve the children throughout the end of life experience.  - Advice and guidance on preparing for the future.  - Advice on keeping key networks (e.g., schools) updated.  - Maximising social networks as and when necessary.  - Making memories and capturing life as it happens.  - Maintaining routines as best as possible.  -Information on the reactions of children to a poor cancer diagnosis.  -Information within the resource provides developmentally appropriate information. | **Needs of parents when dying** ^5-11^  - Advice and guidance on how to prepare and involve children for the dying experience.  - Maximising support networks where available.  - Advice on keeping key networks (e.g., schools) updated.  -Information on the children’s understanding of death/dying.  - Information within the resource provides developmentally appropriate information | **Inclusivity/Anything else relevant to note** (e.g., translated to another language, cultural/ethnic aspects, families in deprivation, families from non-traditional family units) |
| --- | --- | --- | --- | --- |

## Content Analysis graphs from the extraction matrix


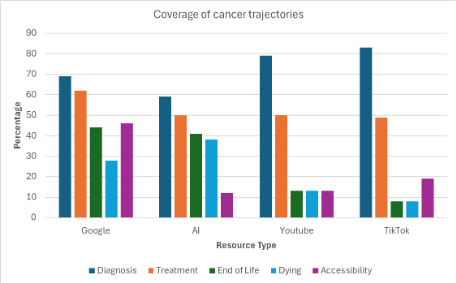

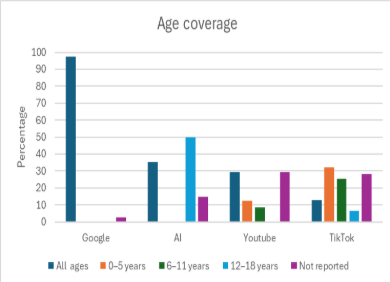


1. Cancer trajectory & Inclusivity B) Age coverage


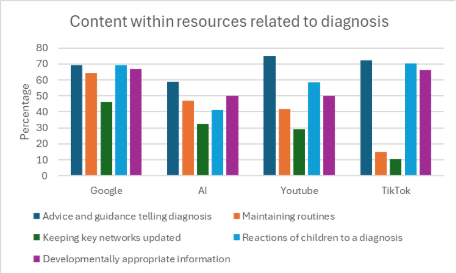

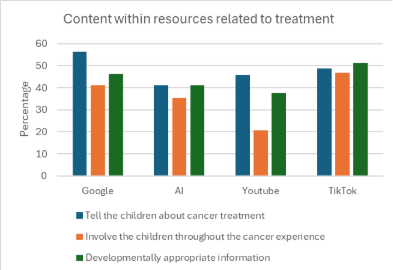


C) Needs of parents at diagnosis D) Needs of parents during treatment


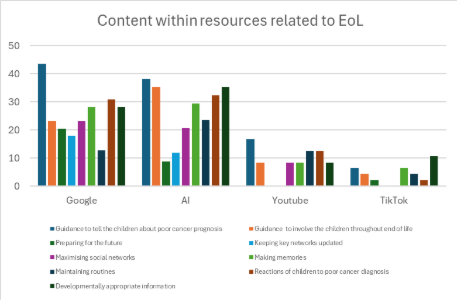

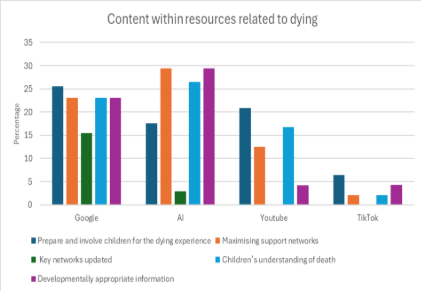


E) Needs of parents at end of life F) Needs of parents when dying

### References:

**Diagnosis**:

1. Semple CJ, McCance T. Parents' experience of cancer who have young children: a literature review. Cancer nursing. 2010 Mar 1;33(2):110-8.
2. Semple CJ, McCaughan E. Family life when a parent is diagnosed with cancer: impact of a psychosocial intervention for young children. European journal of cancer care. 2013 Mar;22(2):219-31.
3. Johannsen, L., Frerichs, W., Inhestern, L., & Bergelt, C. (2023). Exploring the perspectives of cancer patients parenting minor children: a qualitative study on family-centered cancer care experiences. *Patient Education and Counseling*, *117*, 107989.

**Treatment**

1. Asbury N, Lalayiannis L, Walshe A. How do I tell the children? Women's experiences of sharing information about breast cancer diagnosis and treatment. European Journal of Oncology Nursing. 2014 Dec 1;18(6):564-70.

**EoL**

1. Hanna JR, Semple CJ, Strutt L. When an adult with significant caregiving responsibilities for children is at end-of-life with cancer: A carer's pre-bereavement and post-bereavement experiences. In Understanding the Grief and Loss Experiences of Carers 2024 Oct 4 (pp. 94-109). Routledge.
2. Semple CJ, McCaughan E, Beck ER, Hanna JR. ‘Living in parallel worlds’–bereaved parents’ experience of family life when a parent with dependent children is at end of life from cancer: A qualitative study. Palliative medicine. 2021 May;35(5):933-42.
3. Hanna JR, McCaughan E, Semple CJ. Immediate bereavement experiences when a parent of dependent children has died of cancer: funeral directors' perspectives. Death Studies. 2022 Apr 21;46(4):969-78.
4. Semple CJ, McCaughan E, Smith R, Hanna JR. Parent’s with incurable cancer:‘Nuts and bolts’ of how professionals can support parents to communicate with their dependent children. Patient education and counseling. 2022 Mar 1;105(3):775-80.
5. Hanna JR, McCaughan E, Semple CJ. Challenges and support needs of parents and children when a parent is at end of life: a systematic review. Palliative medicine. 2019 Sep;33(8):1017-44.
6. McCaughan E, Semple CJ, Hanna JR. ‘Don’t forget the children’: a qualitative study when a parent is at end of life from cancer. Supportive Care in Cancer. 2021 Dec;29(12):7695-702.

**Dying**

1. Hanna JR, Semple CJ. ‘I didn't know what was in front of me’—Bereaved parents' experience of adapting to life when a co‐parent of dependent children has died with cancer. Psycho‐Oncology. 2022 Oct;31(10):1651-9.
